# Supplementary material for: Are carbon emissions trading and green financial instruments synergistic? -Comprehensive quantitative research based on content analysis
Source: PLoS One. 2024 Mar 7;19(3):e0298601. doi: 10.1371/journal.pone.0298601 (PMC10919601; doi:10.1371/journal.pone.0298601)
Supplement: S1 Data — (ZIP) [file pone.0298601.s001.zip › Data/地级市Z_F工作报告.zip!/╔╜╢1⁄2/╝├─■╩╨/└┤╫╘╣1⁄2╓┌║┼í╛┬φ┐╦╩2╛▌═°í┐_2011.docx]

各位代表：

　　现在，我代表济宁市人民政府向大会报告工作，请予审议，并请市政协委员和其他列席会议的同志提出意见。

　　一、“十一五”规划目标圆满完成，济宁发展站在新的历史起点上

　　过去的五年，是济宁发展史上极不平凡的五年，是我们经受严峻考验、加速经济转型、奋力跨越发展的五年，也是经济实力大幅提升、社会事业全面进步、城乡面貌显著变化、人民群众得到实惠最多的五年。五年来，在中共济宁市委的坚强领导下，全市上下牢牢把握科学发展跨越发展总基调，紧抓经济战略转型不放松，有效应对国际金融危机严重冲击，不断战胜各种困难挑战，开创了又好又快发展的新局面，“十一五”规划目标顺利实现，市十五届人大三次会议确定的年度任务全面完成，改革开放和现代化建设取得重大成就。

　　（一）综合实力跃上新的台阶。预计2010年全市地区生产总值完成2550亿元、比上年增长13%，比2005年翻一番；财政总收入337亿元，地方财政收入169.3亿元、增长25.7%，均为2005年的2.5倍；规模以上固定资产投资1410亿元、增长22.5%，五年累计完成4420亿元，是“十五”时期的2.2倍。社会消费品零售总额963亿元、年均增长19%。城市居民人均可支配收入19650元、农民人均纯收入7250元，年均分别增长12.8%和11.9%。金融机构年末存款余额2256.3亿元、贷款余额1366.4亿元，比“十五”末增加1316.7亿元和743.3亿元。我市经济综合实力得到全面提升。

　　（二）转型发展迈出坚实步伐。在工业领域，五年技改投入1400亿元，200个亿元以上产业大项目建成投产。制造业增加值连续三年超过煤电产业，占规模以上工业比重提高到61.7%。装备制造、能源工业总量突破千亿元，利税超千万元企业发展到350家，太阳、华勤、如意、小松山推及山推股份5家企业销售收入过百亿。在高新技术产业领域，以新能源为代表的战略性新兴产业形成集聚效应，建成五个国家级产业基地。济宁高新区晋升为国家高新区。高新技术产业产值占比由2005年的22.4%提高到35.5%。累计创出11个中国驰名商标、90个省著名商标和144个全国全省名牌产品。在服务业领域，全市服务业增加值年均增长15%，贡献份额提高到33.6%。一批新兴服务业大项目综合带动效应逐步显现，文化旅游资源整合开发深入推进，获省批准设立“尼山省级文化旅游度假区”。去年接待中外游客2830万人次，旅游社会收入214亿元、增长16%。新引入商业银行4家。民营经济上缴税金173.5亿元，五年增长2.2倍。在农业领域，粮食生产连续7年增产丰收。农产品 “三品一标”认证819个，标准化、规模化种养基地增加到350万亩。市级以上产业化龙头企业发展到616家。林业六大工程生态经济效益同步提升。精品渔业品牌竞争力增强。动植物疫病防控和质量检测监管体系日益健全，畜牧业持续稳定发展。完成194座水库除险加固和一批骨干河道治理任务。基本实现农业生产全过程机械化。结构趋于合理的产业格局正在加速形成。

　　（三）改革开放实现较大突破。农村综合配套改革深入推进，集体林权制度改革取得阶段性成果，“镇园合一”管理体制逐步到位。完成50家市属困难企业改制脱困任务，地方煤矿企业整合取得重要进展，国有资本经营预算制度有效实施。太阳纸业、如意科技成功上市。新一轮政府机构改革顺利完成。先后7次压减行政审批项目714个，涉企收费“一费制”改革经验在全国推广，公共交易平台运作初见成效。逐步理顺市县财政收入分配体制，驻济外地煤矿企业实现属地注册纳税，市县投融资平台融资能力不断增强。境内外招商活动成果丰硕，对外贸易规模稳步提升。去年引进市外国内资金409.3亿元，新批办外资项目75个。五年累计直接利用外资20.3亿美元，外贸进出口156.3亿美元，其中出口89.9亿美元，分别是“十五”的2.1倍、2.9倍和2.8倍。29个世界500强投资项目落户济宁。境外投资企业发展到82家。改革的不断深化和开放的日益扩大为我市经济发展注入了蓬勃的生机与活力。

（四）城镇开发建设进程加快。五年累计投入363亿元完成141项重点城建和交通基础设施工程，中心城区建成区面积增加到101.3平方公里、人口发展到93.6万，全市城镇化率提高到43%。编制完成新一轮城市总体规划和土地利用规划。调整优化市中区、任城区行政区划。北湖新城进入全面开发、整体推进新阶段。济北新区公共设施和一批重点工程提速推进。东部科技新城各类先进要素加快集聚，服务功能显著提升。老城区洸河路、共青团路及太白楼路、运河路等主干道得到整体改造，完成212条背街小巷综合整治，全民健身广场、人民公园等公共设施建成开放，供水供气供热管网覆盖面积扩大、保障能力增强。城区管理体制进一步理顺，行政综合执法效能不断提高。以重大工程牵动济兖邹曲嘉五大板块融合发展，基础设施、产业项目、生态建设加速对接、协调推进。济广高速济宁段及6条高速连接线竣工通车，京沪高铁曲阜站、济徐高速济宁段和济宁北外环路正加紧实施。新增农村公路3800公里，城乡公交一体化发展格局初步形成。济宁机场开通6条国内航线。内河通航里程增加到1100公里。南水北调东线系列项目加速推进，治淮东调南下工程基本完成。县域城市旧城改造力度加大、新区建设各具特色，小城镇集约发展步伐加快。积极推进农村土地整治和城乡建设用地增减挂钩试点，五年新建新型农村社区268个。城乡面貌焕然一新。

　　（五）生态环境质量稳步提升。“十一五”节能减排控制目标全面完成。探索出“治用保防控”结合的治污减排新模式，完成76个工业点源深度治理项目，淘汰143家企业落后产能，改造提升13处城市污水处理厂及配套管网，新建7处生活垃圾处理场，7处截污导流工程投入使用。培育8家循环经济先导示范企业，工业“三废”利用率提高到92%。建成环保在线监测平台，62家重点排污企业实现稳定达标排放，主要河流断面全面消除劣五类水质。2007年代表山东接受国家淮河流域治污检查考核获得第一名。33家燃煤电厂完成脱硫改造，秸秆禁烧长效机制基本形成。启动环南四湖大生态带规划建设，建成5个省级国家级生态示范区，种植修复人工湿地15万亩。治理采煤塌陷地5.6万亩。森林覆盖率提高到27%。广大百姓都能感受到济宁山山水水的可喜变化。

　　（六）改善民生和社会建设成效显著。市级财政五年累计民生投入66.7亿元。市政府连年承诺为民所办10件实事如期完成。城镇年均新增就业10万人左右，城镇登记失业率控制在4%以内。累计转移农村劳动力57.7万人次。我市进入首批创建国家级创业型城市行列。养老、医疗、工伤、失业、生育保险参保人员五年净增268万人。连续调高企业最低工资标准、城乡低保标准和“五保”供养标准，及时对受灾困难群众实施救助。建成市社会福利中心。改造扩建乡镇敬老院145处，农村“五保”集中供养率提高到80.2%。残疾人康复、扶贫、就业系列扶持项目有效实施。慈善机构、红十字会在扶贫济困中发挥了积极作用。城镇居民医疗保险基本实现全覆盖。新农合参合率提高到98.7%。新型农村社会养老保险试点工作稳步推进。完成143处乡镇卫生院改造、3838处村卫生室和社区卫生服务中心规范化建设任务。建成经济适用房和廉租房2万余套、149.4万平方米。资助1.3万户农村困难家庭完成危房改造。免费义务教育全面实现，高中阶段入学率提高到90%，职业教育稳步发展，高等教育毛入学率达到26%。改造中小学校舍危房260万平方米，省市级规范化学校增加到468家，农村教师工资实现县级统筹。济宁师专晋升本科院校。济宁医学院、山东理工职业学院北湖新校区投入使用。济宁市技术学院改建为济宁市技师学院。连年成功举办中国专利高新技术产品博览会及“院士济宁行”活动。五年取得省级以上科技进步奖129项，授权专利1.2万件，“如意纺”技术获得国家科技进步一等奖。我市成为“国家知识产权试点城市”。国际孔子文化节影响力持续提升。中华文化标志城规划建设取得积极进展。一批文艺精品在全国、全省获奖。全民健身活动广泛开展，济宁获得第23届省运会承办权。人口自然增长率稳定在6‰左右。第六次人口普查顺利完成。工商、物价、质量技术监督和食品药品监管富有成效。对口援建北川项目提前竣工，新一轮援疆工作全面展开。社会事业的繁荣进步使人民群众共享到更多的改革发展成果。

（七）民主法制和精神文明建设进一步加强。主动接受人大依法监督、政协民主监督，认真办复人大代表建议和政协提案。依法行政“四五”规划和“五五普法”深入实施。政务公开制度日益完善。行政审批服务质量持续提升。顺利完成第九届村民委员会换届直选。行政问责、效能监察和审计监督力度加大，惩治和预防腐败体系建设扎实推进。15个单位获得全国文明单位和文明创建先进单位称号，我市跻身省级文明城市行列，实现全国双拥模范城“五连冠”。治安防控体系日益健全，严打整治专项行动富有成效。社会应急管理和处置公共突发事件能力不断提高。矛盾纠纷排查化解措施到位，信访案件责任制有效落实。“市长公开电话”及时解决了一大批群众反映的热点难点问题。联手创建鲁苏和谐稳定边界、建立医疗纠纷第三方调处机制等经验在全国推广。我市被评为“全国社会治安综合治理优秀市”。安全生产形势稳定好转。国防动员、人民防空、民兵预备役和国家安全工作成效明显。广播影视、新闻出版、民族宗教、外事侨务、地震、气象、水文、档案、史志、文博、老龄和妇女儿童等项事业取得新成绩。

　　各位代表！“十一五”发展成就来之不易，归功于省委、省政府和市委的正确领导，得益于历届领导班子奠定的良好基础，离不开市人大、市政协的监督支持，凝结着广大干部群众的心血汗水。在此，我代表市人民政府，向全市工人、农民、知识分子和干部，向各民主党派、工商联、人民团体和各界人士，向中国人民解放军驻济部队指战员、武警官兵和公安干警，向中央、省驻济单位，向来济宁兴业发展的投资者和建设者，向关心支持济宁现代化建设的港澳台同胞、海外侨胞和国际友人，表示衷心感谢和崇高敬意！

　　过去五年，我们不仅取得了巨大的发展成就，而且积累了丰富的实践经验。一是必须聚精会神抓发展。始终坚持推进科学发展跨越发展不动摇，大胆解放思想，提升发展境界，切实强化又好又快的政策导向、利益导向和考核导向，瞄准更高目标定位，奋力实现跨越赶超。二是必须坚定不移促转型。准确把握市情特点和发展阶段性特征，自觉运用国际金融危机和新科技革命形成的倒逼压力，集中打好转方式调结构攻坚战，推动经济增长与转型升级互促并进。三是必须统筹兼顾求协调。注重从政策扶持、投入倾斜、制度创新、监督考核等层面抓起，推动城乡一体化发展，促进社会事业全面进步，加速公共服务均等化步伐，持续改善生态环境质量。四是必须锐意创新增活力。主动推进重点领域、关键环节的改革，不断改进完善管理体制、运营机制和工作模式、领导方法，真正形成有利于科学发展跨越发展的制度框架；切实提高利用“两个市场”、“两种资源”的能力，借助外力增强济宁经济的整体素质和国际竞争力。五是必须以人为本惠民生。不折不扣兑现落实各项惠民政策，扎实推进创业富民、就业惠民、社保利民、稳定安民，每年集中为城乡百姓办一批实事好事，让人民群众更多地享受改革发展成果。六是必须改进作风重实干。严格落实“三重”工作目标责任制，全面推行指挥部会战体制和“一线工作法”，持续营造求真务实、苦干实干的环境氛围，鼓励引导广大干部拼搏奉献、克难攻坚、创优争先。这些经验弥足珍贵，是指导和推动新一轮发展的宝贵精神财富。

　　同时我们清醒地看到，全市经济社会发展还存在不少困难和问题，政府工作也有一些差距和不足。主要是：经济结构性矛盾仍较突出，新兴产业竞争优势不明显，服务业发展滞后；自主创新能力不强，经济外向度偏低；农业产业化经营水平不高，农民持续增收基础不稳固；资源约束加剧，环境压力加大；县域发展不平衡，部分县乡财政实力较弱；城市化水平提升不快，中心城区辐射带动功能尚未充分发挥；社会建设存在薄弱环节，改善民生促进社会和谐的任务艰巨；政府职能转变还不到位。我们要正视问题、找准症结、切实改进，努力把各项工作做得更好。

二、围绕“十二五”发展目标，扎实做好开局年的各项工作

　　“十二五”时期是我市打造区域发展新高地、实现富民强市新跨越的关键时期。按照中央《建议》和省、市委部署要求，市政府编制了《济宁市国民经济和社会发展第十二个五年规划纲要（草案）》，在系统总结“十一五”发展成就与经验，透彻分析未来五年发展大势与宏观环境的基础上，提出了“十二五”全市经济社会发展的总体要求、目标任务和主要政策措施。本次大会审查批准后，我们将分解目标，明确责任，认真组织实施，确保各项任务落到实处。

　　今年是实施“十二五”规划的开局之年。我们既面临京沪高铁全线开通、南水北调工程提速、23届省运会加紧筹办、沿海发达地区产业转移步伐加快等诸多难得机遇和有利条件，又面临外部环境复杂多变、宏观调控政策趋紧、区域发展竞争加剧等方面的压力挑战。做好今年工作，要按照省、市委的部署要求，以科学发展为主题，以加快转变经济发展方式为主线，以富民强市为目标，高扬科学发展跨越发展总基调，突出重点、突出特色、突出创新，在调整优化产业结构、加快城镇化进程、提高自主创新能力、强化节能减排和生态保护、推动体制机制创新、保障改善民生等方面实现新突破，加速现代产业新城、文化旅游名城、生态宜居水城建设，保持经济平稳较快发展，促进社会和谐稳定，确保“十二五”开好局、起好步。经济社会发展主要预期目标是：地区生产总值增长12%，地方财政收入增长15%，全社会固定资产投资增长20%，社会消费品零售总额增长16%，外贸进出口和实际利用外资均增长10%，城市居民人均可支配收入和农民人均纯收入均增长12%，城镇登记失业率控制在4%以内，居民消费价格涨幅控制在4%，完成省下达的节能减排年度约束指标，人口自然增长率控制在7‰以内。着力抓好十个方面的工作。

　　（一）做大做强工业经济。着眼高端高质高效目标，创新转型发展路径，集中突破重点领域和关键环节，力促工业结构优化升级，显著提高整体素质和竞争实力。

　　加快传统产业改造提升。深入实施“千户企业改造提升工程”， 广泛运用和消化吸收先进实用技术，突出抓好75项省级重点技改项目，年内全市技改投资完成500亿元、占工业投资的比重达到60%以上。启动“利税千万元企业信息化改造”工程，着力推进技术装备、生产制造、经营管理、营销物流的数字化和信息化。与有关高校联手打造工业设计平台，为企业提供工艺、流程和产品设计服务。开展争创省长、市长质量奖活动，年内新增中国驰名商标2个、省著名商标12个和省名牌产品10个。

　　扶持新兴产业成长壮大。强化规划引导和政策倾斜，完善财税金融扶持政策，引导更多要素向新能源、新材料、生物医药、信息技术和节能环保产业等五大领域聚集。推广引入台联电、建设光电信息产业园的成功模式，突出抓高端大企业、大项目的引进建设，支持一批重点企业研发新产品、转化新成果、扩张优势产能，同步跟进相关配套协作项目，尽快形成核心竞争力强、产业链条完整、规模效益突出的新兴主导产业。年内战略性新兴产业销售收入增幅力争突破35%、总量达到350亿元。

　　推动主导产业集约集聚。深入对接国家、省产业调整振兴规划，积极争取项目布局和资金投入的倾斜，促使煤化工和机械制造、纺织新材料、生物技术、专用汽车、光电特色产业等国家级产业基地提升规模档次。推进化工、食品两大市级重点园区开发建设，持续增加投资强度和项目密度，加强关联企业和上下游产品组合协作，构建完整产业链条。着力抓好 “创百亿工程”，支持优势企业通过资本运作、技改扩能、合资合作、多元经营、挂牌上市等途径壮大实力、晋档升级，争取再有一批企业销售收入突破50亿、100亿，现有百亿企业加速向300亿、500亿迈进，新增3—5家上市企业。

　　（二）构建现代农业产业体系。强化农业基础地位，围绕实现生产经营规模化、标准化、专业化、集约化，深度调整农业结构，加快转变农业发展方式，提高综合产出效益，促进农民持续较快增收。

　　壮大特色主导产业。严格耕地保护，稳定粮食面积，实施良种工程，加快中低产田改造，开展粮棉油高产创建活动，力争全年粮食总产突破100亿斤。优化种养结构布局，扩大优质、高效、生态、安全的设施瓜菜生产规模，确保供给充足、价格平稳。按照“一镇一业、一村一品”格局，扩张发展大蒜、食用菌、林果、特色畜禽、精品渔业、苗木花卉、中药材等特色产业，尽快形成支撑区域现代农业体系的专业基地和产业带。健全农业技术推广、动植物疫病防控和农产品质量监管体系，在特色基地和各类农业园区内全面推行标准化生产，继续抓好无公害、绿色、有机和地理标志农产品认证及品牌推介活动，支持济宁农高园争创国家级农高园。年内全市高价值经济作物扩大到330万亩，农产品质量安全示范区发展到250处，“三品一标”认证达到900个。

　　提升产业化经营水平。以特色产业延伸链条、优势农产品加工增值为目标，鼓励引导资本、技术、人才等要素进入产业化经营领域，高起点创办龙头项目。扶持发展各类专业化合作经济组织，提高农业现代化组织与服务能力。密切农户、专业园区与加工企业、批发市场的利益联结，创办“绿色车间”、发展“订单农业”，支持特色基地和园区与大中城市商场、超市开展“农超对接”。年内新增规模以上农业产业化龙头企业50家、农村合作经济组织和专业协会100个。

　　强化农业基础支撑。抓住国家启动新一轮水利建设和山东创建现代水利示范省的有利时机，争取更多水利工程列入国家、省“大盘子”。全面完成治淮东调南下工程，加速推进南水北调系列项目，适时启动引汶入济、引黄调水工程。加紧实施去冬今春4638项农田水利项目，抓好泗河等骨干河道生态化治理，完成8个全国小型农田水利重点县建设任务，有序推进大型灌区续建配套和末级水系改造工程。全面展开省、市农村土地综合整治示范工程项目。加快小流域综合治理步伐，推动灌溉、道路、电力等基础设施向山区、湖区、滩区种养基地、专业园区延伸覆盖。建立农村公路管理养护长效机制，年内改造农村公路400公里、桥梁30座。稳定提升全市农业机械化综合水平。

　　拓宽农民增收渠道。落实配套资金，保证各项惠农补贴及时足额到位。引导农民调整种养结构、大搞设施农业，靠农业产出效益的提高增加收入。建立健全城乡一体的就业指导服务网络，强化农民技能培训，定向开发劳务输出基地，疏通农民就地转移就业渠道，鼓励扶持返乡创业，持续增加工资性和财产性收入。积极实施开发式扶贫和大中型库区移民后期扶持项目。继续做好减轻农民负担工作。

（三）创新发展现代服务业。把服务业特别是现代服务业作为重要的支柱产业，加大资金投入、强化项目支撑、拓展经营领域、完善扶持政策，促其发展不断提速、结构更加优化、贡献份额明显提升。年内服务业增加值占GDP的比重提高2个百分点以上。

　　突出发展重点。文化旅游方面，充分利用我市得天独厚的优势，统筹孔孟文化、运河文化、水浒文化、佛教文化和微山湖生态文化等各大板块的文化旅游资源，形成关联效应和集群优势，集中抓好尼山圣境、明故城、兴隆文化园、水浒文化主题公园等一批重点项目，继续完善重点景区景点配套设施与服务功能，强化精品线路整合和重点客源市场促销开发，力争年内旅游社会收入突破260亿元、增长20%以上。现代物流方面，加快豪德商贸城、医药物流园及港区配套设施建设，加紧实施铁路客货站点改造及铁水联运物流项目，规划建设济宁运河物流中心，积极发展内河集装箱运输和临港物流产业。金融服务方面，鼓励济宁银行跨区域设立分支机构，支持农信社改制组建农村商业银行，协调推动1—2家股份制商业银行入驻我市。促进证券、保险业稳定健康发展。商贸服务方面，加快建设以太白楼路为轴心的中央商务区，引进大连万达等知名商务企业，培育发展总部经济。支持县域城市实施中心商圈项目，兴建各具特色的美食、购物、休闲“一条街”。积极培育健身、医疗、养老、家政及社区服务等新的消费热点，发展电子商务、连锁配送、物联网、移动多媒体等新兴项目，扎实推进“数字城市”建设。房地产方面，继续办好房地产博览会及推介会，吸引大型房地产商来我市投资开发，支持市内房地产企业壮大实力。深入推进“万村千乡市场” 工程。

　　构建发展载体。依托曲阜国家级文化产业示范园等重点园区，深度开发特色文化产品，集中引进影视、广告、形象设计等创意文化项目。依托京沪高铁曲阜站规划兴建文化生态新城，迎接“高铁时代”到来。全面扩大对外开放，发展与海内外知名旅游企业的联合协作，支持市内工商企业、旅行社联手打造大型旅游企业集团。以文艺团体转企改制为突破口，加快文化产业市场化步伐，尽快组建新闻出版、传媒、影视、演艺等文化企业集团。支持济宁高新区等重点园区兴办软件业，建设服务外包产业基地。

　　强化政策扶持。在市场准入、土地、税费、信贷等方面，研究制定扶持服务业发展的优惠政策。充实服务业发展引导资金，集中支持重点区域、重点园区、重点企业的大项目建设。健全服务业评价指标体系，增加服务业考核权重。出台优惠政策吸引专门人才和专业团队，来我市领办创办高端服务业项目，参与商贸企业、大型场馆、重点节会活动的开发运营，走出运用市场机制盘活存量资产、打造服务品牌新路子。

　　（四）切实增强开放招商实效。区域经济的持续繁荣发展，必须建立在市场高度开放、要素流动活跃的基础之上。要大力构筑全方位、宽领域、多层次的开放格局，加速我市经济与外部资源、市场的对接融合，为转型发展增添后劲和动力。

　　创新完善招商方式。面向重点国家和地区，精心包装推介项目，下功夫搞好专案策划和先期对接，有针对性地组织境内外系列招商活动。采取灵活方式，突出抓好专题招商、企业招商、节会招商、园区招商和产业链招商。重点加强与世界500强、跨国公司及国内高科技园区、大型央企和民营巨头的合资合作，在战略性新兴产业、现代服务业、城市开发建设等领域招大引强上率先突破，争取引进一批投资过10亿、50亿以及超百亿的大项目。

　　着力优化投资环境。在环评、土地、审核、立项等环节优化程序、规范操作、提高效率，确保招商项目依法合规运作落地。贴紧大项目引进建设需要，在新能源、装备制造、化工食品等领域先期创办一批产业关联度高、创新优势突出的工业设计中心，最大限度降低投资运行成本。继续实行重点项目目录管理、领导包保和指挥部会战体制。组织开展银企对接及项目推介活动，争取更多信贷资金投入项目建设。强力净化市场环境，严厉打击各种排斥外来竞争、恶意垄断市场的不法行为。

　　培育外贸竞争新优势。调整优化进出口商品结构，提高农副产品、纺织服装等传统出口商品附加值，增加先进制造业特别是高新技术产品出口比重，加强机械电子、生物科技、汽车及零部件等出口创新基地建设，培育出口大企业大集团。积极推进出口市场多元化，深度挖掘欧美、日韩、港澳台等传统市场潜力，努力扩大东南亚、拉美、非洲、俄罗斯等新兴市场占有份额。鼓励企业优先进口重要原材料和急需先进设备、关键零部件。支持海关、检验检疫等部门联手推进“大通关”建设。鼓励有条件的企业“走出去”，实施海外并购、开发战略资源、建立生产基地和营销网络。深化区域战略合作，扎实推进援疆系列项目。

　　（五）进一步强化科技创新与人才支撑。深入实施科教兴市和人才强市战略，全面落实科技人才优先发展、优先投入的政策举措，推动国家创新型试点城市建设取得新进展，增强转型发展的创新驱动力。

　　抓好创新平台建设。实施“四个一百”和“1441培基”工程，鼓励优势骨干企业联手大院大所创办更多高层次研发机构。支持济宁高新区推进“二次创业”，加快建设数控技术研发中心、国家LED质检中心等平台，构建高层次研发服务体系。确保鲁南工程技术研究院年内建成运行，提升鲁南煤化工研究院等平台装备水平与研发能力，争取承担更多国家、省重大科技专项。年内新建省级以上企业技术中心、工程技术研究中心5家，重点实验室和院士工作站5家，科技企业孵化器面积增加到100万平方米。

　　多方增加科技投入。建立完善“政府引导、企业主体、市场运作、多元集聚”的科技投融资体制。今年市级财政继续增加科技人才投入。积极争取更多国家、省科技专项资金。对自主研发和成果转化成效显著的企业加大 “以奖代补”力度，高新技术企业、科技型企业研发经费占销售收入的比重不低于5%。推进科技与金融资本深度融合，鼓励引导更多信贷投入支持创新发展，探索风险投资、私募基金、科技板块上市等融资新途径。年内全市新增国家级高新技术企业20家，高新技术产业产值增长30%以上、占比提高2个百分点以上。

　　着力打造人才高地。把加强人才队伍建设作为强市之基、竞争之本、转型之要，全面落实“中长期人才发展规划纲要”，加强完善人才培养、引进与使用的政策举措。各级要加大人才培养、引进和奖励的投入力度，以重点人才工程为抓手，深入实施海外人才引进“511”计划和国内人才集聚 “百千万”工程，集中培养引进高层次科技人才、高水平创新创业团队、高素质管理人才。借助合作创新联盟的人才优势，在重点行业和领域开展联合攻关，突破制约产业升级的技术瓶颈。加大国外智力引进力度，增强“院士济宁行”活动实效。扎实推进“国家知识产权示范城市”创建工作。发挥驻济高校和职业技术院校优势，实施“金蓝领”培训工程，为重点行业、骨干企业定向培养技能型实用人才。规范完善技术、管理等要素参与分配的激励政策，鼓励支持学科带头人、专利持有人来我市创业发展。继续重奖创新创业贡献突出的优秀人才。一个百川归海、人才辈出的济宁，必将是充满活力和希望的济宁。

（六）大力实施城镇化追赶战略。坚持既定城市发展战略定位不动摇，以总体规划为引领、重大工程为牵动，做优做大中心城区，促进组群结构城市深度融合，协调推进县域城市和小城镇开发建设，加快城镇化进程。

　　大力推进中心城区开发建设。按照“保续建、保重点、保民生” 的要求，有序展开“一城四区”建设项目。突出抓好北湖新区路网完善，加快市文体中心、省运会指挥中心、规划展示馆和济宁一中新校区等重点工程建设进度，精心实施中心景区打造、古运河南段及新运河整治和沿岸开发，同步推进住宅、医院、商贸服务区的配套建设，初步展现生态新城靓丽形象。加快济北新区行政、商业、文化、物流四大功能分区建设，继续延伸拓展城区北二环路，完成新建改建主干道的景观绿化，兴建各具特色的集中居住区。加快建设东部科技新城创新大厦、佳世客商业中心和产学研基地等重点工程，高标准完成洸府河、蓼沟河综合整治及景观带打造，实现与任城开发区、兖州开发区基础设施的对接连通，协调推进高端产业园区与公共服务设施的配套完善，形成创新型经济发展与城市形象提升的良性互动。有序推进老城区重点片区改造，配套完善公共设施，优化主干道路网结构，基本完成背街小巷整治任务。加大企业“退城进园”和城中村改造力度，进一步疏解城市空间、增强服务功能、优化人居环境。

　　促进济兖邹曲嘉都市区融合发展。以重大基础设施为牵动，以园区建设、重点工程、产业项目为支撑，实施交通设施一体化建设、产业市场一体化布局、生态环境一体化整治、人才科技一体化保障，加速五大板块的相向发展。突出抓好京沪高铁曲阜站基础设施配套完善，科学实施高铁片区整体开发，建设高铁站与中心城区、周边县市区“放射状”连接通道。按时间节点完成邹城—嘉祥快速干线、济徐高速济宁段等建设任务。加快推进京杭运河济宁至东平湖段航道北延工程。及早兴建滨湖大道北湖—鱼台段。科学布局重点园区和主导产业，引导相关县市区集中实施先导性大项目，打造特色产业集群。

　　统筹抓好县城和镇村规划建设。坚持旧城改造与新区开发并重，配套完善县城道路交通、给排水、供热供气、污水垃圾处理等基础设施，提高整体承载能力。坚持依法拆迁、和谐拆迁，切实维护群众合法权益。抓好14个新型乡镇和一批省级中心镇建设，按照工矿型、商贸型、农产品加工型、交通枢纽型、旅游观光型等不同模式，打造人口集聚、产业集群和商品集散的中心，增强经济强镇的辐射带动能力。尊重农民意愿，坚持分类指导，稳妥有序推进新型农村社区建设。

　　加强改善城市管理。以创建国家园林城市为契机，加紧实施改造建设系列项目，深入开展城乡环境综合整治，新建一批城市公厕，探索建立城乡保洁长效机制，促使市民文明素质与城市品位形象同步提升。科学调控城区交通，落实公交优先措施，切实解决部分路段拥堵问题。加快构建大城管格局，明确各层级管理责任，突出“四区”主体地位，发挥街道、社区基础作用。强化规划刚性约束，严厉查处各类违法违规建设行为。以数字化城市管理监督指挥中心为平台，加快建立网格式、精细化监管体系。

　　（七）推动县域经济跨越赶超。县域经济是区域经济的重要支撑，与壮大中心城区综合实力有着同等重要的意义。要健全促强扶弱激励导向机制，加大财政转移支付，在基础设施、产业项目、社会建设等方面向经济薄弱地方倾斜，促使经济强市持续争先，中间县市奋力赶超，欠发达县负重崛起，尽快扭转县域之间差距拉大、财力失衡的局面。

　　放手发展民营经济。进一步落实优惠政策、放宽准入领域，鼓励民间资本全面进入三次产业，支持民营企业参与国有企业改制重组。调整优化民营经济布局结构，引导骨干企业向园区聚集，支持优势企业大搞合资合作和技术改造，鼓励中小企业走好专、精、特发展路子，培植更多民营经济特色产业集群。

　　打造特色竞争优势。依托资源禀赋，发挥比较优势，实施错位发展战略，选准壮大特色产业的着力点和突破口，在农产品精深加工、大工业配套协作、制造业晋档升级、文化旅游资源开发等领域加大要素投入、兴办特色项目，支持有条件的县乡引进转化最新技术成果，培育发展高新技术产业和新兴服务业。年内各县市区新增规模以上企业30个以上，引进建设千万元以上项目50个以上。

　　切实增强可用财力。强化工业主体支撑，千方百计做强企业、做优产业、做大财税增收“蛋糕”。找准国家产业政策、财税政策与县乡基础优势的结合点，集中扶持税收贡献大、地方留成多的企业和项目。依法强化税收征管，完善社会综合治税工作机制，努力提高“两税”收入占比。

　　（八）着力建设生态文明。生态环境关系百姓福祉，关系未来发展。要坚持环保优先、节约优先，严格落实节能减排目标责任和关键举措，加快构建资源节约、环境友好的生产方式和消费模式，推动生态城市建设，把蓝天碧水还给广大人民群众。

　　突出抓好节能减排。继续实施重点节能工程，着力在工业、建筑、交通运输、公共机构等领域推广节能新工艺、新设备、新材料。实施新一轮淘汰落后产能计划，年内再淘汰立窑水泥65万吨、烧碱6万吨及一批高耗能设备。制定《济宁市2011年度污染物减排计划》，进一步完善减排控制指标体系。加紧实施工业点源深度治理项目，如期完成城市污水处理厂升级改造，新建一批生活垃圾处理场，年内确保中科济宁垃圾发电厂建成发电。严格控制农业面源污染。强化废水排放监测预警，开展经常性环保执法专项行动，实现主要河流断面水质稳定达标，确保淮河治污迎查取得优异成绩。深化燃煤电厂脱硫脱硝改造整治，全面取缔城区小型燃煤炉具，强化机动车尾气污染治理，有效控制建筑扬尘污染，巩固扩大异味污染企业整治成果。

　　大力发展循环经济。积极争取国家、省循环经济发展专项资金，推进低碳减排技术创新和产品开发利用。扩充循环经济先导示范企业、示范园区群体，全面实施清洁生产和环境管理体系认证，引导骨干企业和重点园区率先构建绿色产业链和资源循环利用链。推进清洁能源、可再生能源和新能源的有序发展、有效利用，推广应用LED新光源、电动汽车等先导产品。面向发电企业及部分行业和城市绿化、保洁等领域大力推行中水回用。

　　强化生态恢复重建。加快实施环南四湖大生态带建设，严格各类生态功能区、自然保护区建设管理。多措并举治理采煤塌陷地。实行最严格的水资源管理制度。开工3个生态修复和人工湿地水质净化项目，新建人工湿地1万亩。全面推进新一轮林业建设六大工程，年内森林覆盖率提高1个百分点。

（九）稳步推进体制机制创新。改革未有穷期，改革才有活力。要着眼破除一切影响制约科学发展跨越发展的制度障碍，突出抓好重点领域关键环节的改革，为经济社会发展创造良好的制度环境。

　　加快国有企业改革步伐。因企制宜加紧实施国有企业股份制改造，健全完善法人治理结构。鼓励引导骨干企业开展兼并重组，实现产权多元化。支持优势国有企业做强主业、拓展领域、进军新兴产业。进一步强化国有企业业绩考核和薪酬分配激励约束，加快推行外部董事和外派监事会制度。

　　继续深化农村改革。在依法自愿有偿基础上，健全土地承包经营权流转市场运营机制。尽快完成集体林权、土地经营权和宅基地、养殖水域、小型水利工程使用权的确权发证。积极培育村镇银行、贷款公司和资金互助社等农村金融组织。进一步完善“镇园合一”管理体制。

　　扎实推进社会领域改革。抓好医药卫生体制改革，全面推行、不断完善基本药物制度，搞好公立医院改革试点，加紧实施基本公共卫生服务均等化项目。稳妥实施教育体制改革，推行中小学校长任职资格和职级制度，创新中学生学业水平考试和综合素质评价制度办法，探索建立政府指导、企业参与、学校自主办学的职业教育合作发展机制，完善教师培训培养模式与职称评聘制度。深化户籍制度改革，鼓励有条件的农民到城镇购房、定居和创业发展，同步完善落实社保、教育、医疗等方面的配套政策，保证进城农民真正享受到市民待遇。

　　加大政府管理体制改革力度。巩固发展政府机构改革成果，尽早启动事业单位分类改革。切实转变政府职能，坚决推行政企分开、政事分开、政府与中介机构分开，集中力量强化社会管理和公共服务。健全土地出让、产权交易、工程招投标、政府采购一体化的公共交易运行与制衡机制，规范发展、严格监管中介服务，克服权力寻租现象，确保公开公平公正。强化财政综合预算管理，将公共资源收益全部纳入预算范围。扩大部门预算覆盖面，细化编制内容，强化刚性约束。

　　（十）加强以改善民生为重点的社会建设。保障改善民生是践行执政为民宗旨的基本要求。要持续加大民生投入和政策扶持力度，优化配置公共服务资源，进一步向基层倾斜、向普通民众倾斜、向弱势群体倾斜。围绕解决关系城乡百姓切身利益和生产生活实际的困难问题，继续承诺办好年度10件民生实事。

　　努力扩大就业。深入落实更加积极的就业政策，扎实推进国家级创业型城市建设，突出抓好高校毕业生就业工作，继续实施“大学生创业引领计划”和基层就业服务项目，统筹做好农村转移劳动力、城镇就业困难人员、退役军人就业工作。年内城镇新增就业7.2万人，高校毕业生初次就业率力争达到85%，实现“零就业”家庭动态消零。严格执行最低工资标准，督促企业建立工资正常增长和支付保障机制，强化劳动监察执法，构建和谐劳动关系。

　　健全社保体系。新农保试点扩大到8个县市区。逐步把城镇非就业居民和下岗失业困难职工纳入基本养老和医疗保险范围。规范完善城镇基本医疗、工伤、失业保险市级统筹。建立社会救助与物价上涨联动协调机制。提高新农合财政筹资和公共卫生服务人均标准，调高工伤人员伤残津贴、护理费和抚恤金标准，年内企业退休人员基本养老金人均增加140元，城市低保标准提高到每人每月260元—320元，农村低保提高到每人每年1400元—1700元，新农合住院报销比例提高到60%以上。积极发展社会福利和慈善事业。加紧建设市残疾人综合服务中心，为1万名残疾人提供康复救助与托养服务。落实“菜篮子”、“米袋子”行政首长负责制，改扩建一批农贸市场。健全“12315”工商行政执法体系，持续加大食品药品监管力度，切实加强饮用水水源地保护。提高气象、地震监测预报水平，增强防灾减灾能力。

　　落实教育优先。制定实施全市中长期教育改革发展规划纲要，持续增加教育投入。加快普及学前三年教育，年内力争城乡幼儿入园率提高到75%以上。深入推进素质教育。调整优化济宁城区中小学布局结构，扩大高中阶段办学规模，建立城乡、强弱校之间校长、教师交流机制，合理配置优质教育资源。实施职业教育基础能力建设工程，年内创建1—2所国家级中等职业教育改革发展示范校。引导济宁学院和高职院校改善装备条件、加强学科建设、彰显品牌优势。支持曲阜师范大学、济宁医学院立足济宁创新发展，建成富有地方特色的知名高校。健全助学体系，不让一个孩子因家庭贫困而失学。

　　改善医疗服务。立足于让老百姓方便看病和看得起病，巩固乡镇卫生院、村卫生室配套建设成果，扶持县级医疗机构改善硬件装备、提高诊疗水平。健全城市社区卫生服务网络，集中建设10个示范服务中心。有机整合区域医疗卫生资源，完善城乡医疗机构对口援助机制。扎实推进“优生优育三级预防工程”和农村育龄妇女专项健康查体工作。重视发展中医药事业。加强传染性疾病、地方病、职业病防治和精神卫生工作，提高重大疾病预防控制和突发公共卫生事件救治能力。

　　强化住房保障。没有百姓的安居，就没有社会的安宁。要大规模推进保障性住房建设，年内开工公租房、经济适用房和廉租房1.4万套、81万平方米。强化县市区和街道、社区的组织实施责任，加快“城中村”、棚户区改造步伐。为农村贫困家庭改造危房3000套，彻底消除“土坯房”。优化住房供给结构，提高中小户型、中低价位住房比重，有效稀释房价上行压力。

　　繁荣先进文化。推进社会主义核心价值体系建设，广泛开展群众性精神文明创建活动。加紧实施文化信息资源共享工程系列项目，提升基层文化设施服务功能。强化文化市场执法监管。繁荣文学艺术创作和哲学社会科学。推进文博、史志、档案服务社会化。引入市场机制，创新运作模式，提高国际孔子文化节和“中国文化遗产日主场城市”活动水平。加紧实施中华文化标志城孔孟文化遗存保护项目。加强国防动员、人民防空、民兵预备役建设，争创全国双拥模范城“六连冠”。加快23届省运会场馆建设进度，同步完善全民健身设施网络。建立健全计划生育联动监管平台，持续稳定低生育水平。统筹做好广播影视、新闻出版、民族宗教、外事侨务和对台工作，保障妇女儿童和老年人合法权益。

　　维护社会稳定。落实维稳第一责任，深入推进“平安济宁”建设。加强新形势下群众工作，创新完善社会管理、矛盾化解、公正廉洁执法三项重点工作举措。严格领导干部公开接访和信访案件包保责任制，建立健全重大工程项目建设和政策制定的社会稳定风险评估机制。完善社会治安防控体系，依法防范和严厉打击各种违法犯罪活动。重视做好国家安全工作。扎实开展“安全生产基层基础深化年”活动，进一步抓好重点领域和行业的专项整治。完善社会应急管理机制，提高预防预警和应急处置能力。

三、全面加强政府自身建设，为科学发展跨越发展提供有力保障

我们正处在一个急剧变革的时代。在未来区域经济版图中占有什么样的地位，在很大程度上取决于我们今天的智慧和勇气。面对复杂形势和繁重任务，我们必须紧紧围绕科学发展主题和转变经济发展方式主线，准确把握大势，锐意改革创新，切实改进作风，凝心聚力实干，不断提高驾驭全局、领导科学发展的能力，不断提高行政效能和服务水平，不断提高政府公信力和执行力，更好地担负起历史的重任。

我们要建设学习政府。大兴学习之风，善于接受新知识、新理念，敏于研究新情况、新问题，以学习引领新的思想解放，以更加宽广的视野、胸襟和思路谋划发展、追求卓越、实现突破。弘扬勇于开拓、敢为人先的精神，坚定革故鼎新、自我超越的意志，不为过去的成绩而自满，不为既有的经验所束缚，不为传统的模式所局限，旗帜鲜明地鼓励创新、支持改革，更大力度推进发展思路、体制机制、政策举措、运作模式的与时俱进，在克难攻坚、锐意进取中创造新优势、开创新局面。今年起在政府机关开展“读一本好书”活动，让学习逐渐成为责任，成为时尚，成为习惯。

我们要建设法治政府。每一个政府工作人员都要满怀对法律法规的尊重与敬畏，切实增强法制意识，努力提高学法懂法守法用法的自觉性。深入落实依法行政实施纲要，严格遵循政府工作规则，严格规范政务运行流程，严格依照法定权限和程序行使职权，在行政诉讼案件中逐步实现行政首长应诉制度。自觉接受人大法律监督和政协民主监督，认真执行人大及其常委会各项决议决定，及时办理人大代表建议、政协提案，加强与工会、共青团和妇联等群团组织的联系。健全重大问题调查研究、专家咨询和社会公示与听证及决策责任制度，保证决策科学民主、依法合规、切实可行。按照公开是原则、不公开是例外的要求，进一步拓展政务公开的领域和内容。加快发展电子政务，高水平建设政府门户网站，务求各项工作阳光运行、公开透明。加强行政执法队伍建设，落实执法责任制和责任追究制，确保公正文明执法。

我们要建设效能政府。倡导敢于负责、勇于担当，鼓励雷厉风行、奋勇争先，力戒形式主义、官僚主义，力戒心浮气躁、急功近利，力戒简单粗放、盲目蛮干。牢固树立正确的政绩观，多做促发展壮实力的事，多做顺民意惠民生的事，多做打基础利长远的事，创造经得起实践、群众和历史检验的实绩。对重大事项、重要决策、重点工作层层分解、明确责任、跟踪督查、严格考核，加大行政问责、效能监察力度。落实服务承诺制、首问负责制、限时办结制，大力推行第一时间服务、零距离服务、全过程服务，引导市县行政审批中心完善机制、高效运行，促进发展环境不断优化。从市政府做起，下决心精简会议文件，摆脱繁琐事务，集中全部心思和精力抓执行、促落实。各级领导干部特别是主要领导要经常深入建设发展最前沿亲历亲为，务求把问题解决在一线、把难点突破在一线，把任务完成在一线。

我们要建设民本政府。心系百姓疾苦冷暖，象抓经济建设一样抓民生工程，把保障改善民生作为政府工作的出发点和落脚点。深入体察民情、顺应群众期待，从柴米油盐、衣食住行、上学就业、求医问药、助老托幼等方面入手，一件一件地抓落实、求实效，尤其要为困难群众、弱势群体“雪中送炭”，让他们充分感受到党和政府的关爱温暖。加大民生实事督查督办力度，确保实事项目如期兑现、取信于民。进一步办好市长公开电话，及时倾听社情民意、化解矛盾纠纷、改进政府工作。

我们要建设廉洁政府。严格遵守廉洁从政各项纪律规定，常思贪欲之害，常除非分之念，常怀律己之心，始终保持党员领导干部的气节和本色。坚持勤俭办一切事情，反对铺张浪费、奢靡之风，带头创建节约型机关。深入落实党风廉政建设责任制，强化对政府部门领导班子、领导干部的教育、管理和监督。在关系人、财、物的重点部位和关键环节完善制度、强化监管、规范行为，从源头上有效预防和治理腐败。进一步加强审计监督，支持纪检监察机关依法依纪查办案件，坚决纠正侵害群众利益的不正之风。每一个政府工作人员都必须把清正廉洁作为牢牢坚持的操守和底线，以为民、务实、清廉的良好形象，赢得广大人民群众的信赖支持。

各位代表！ “十二五”宏伟目标催人奋进，全面小康社会建设任重道远，孔孟之乡圣地沃土生机勃发、大有希望。让我们在中共济宁市委的坚强领导下，紧紧依靠全市人民，振奋精神、锐意开拓，同心同德、拼搏实干，奋力开创济宁科学发展跨越发展的新局面，以优异成绩迎接建党90周年！
